# “No one talks about it”: using emotional methodologies to overcome climate silence and inertia in Higher Education

**DOI:** 10.3389/fsoc.2024.1456393

**Published:** 2024-11-27

**Authors:** Anna Pigott, Hanna Nuuttila, Merryn Thomas, Fern Smith, Kirsti Bohata, Tavi Murray, Marega Palser, Emily Holmes, Osian Elias

**Affiliations:** ^1^Faculty of Science and Engineering, Swansea University, Swansea, United Kingdom; ^2^Centre for Ageing and Dementia Research, Swansea University, Swansea, United Kingdom; ^3^Geography Department, University of Exeter, Penryn, United Kingdom; ^4^Emergence, Powys, United Kingdom; ^5^Faculty of Humanities and Social Science, Swansea University, Swansea, United Kingdom; ^6^Iaith, Carmarthenshire, United Kingdom

**Keywords:** climate and ecological crises, emotional methodologies, emotional reflexivity, climate action, connection, Higher Education

## Abstract

Higher Education (HE) is, at best, struggling to rise to the challenges of the climate and ecological crises (CEC) and, at worst, actively contributing to them by perpetuating particular ways of knowing, relating, and acting. Calls for HE to radically transform its activities in response to the polycrises abound, yet questions about how this will be achieved are often overlooked. This article proposes that a lack of capacity to express and share emotions about the CEC in universities is at the heart of their relative climate silence and inertia. We build a theoretical and experimental justification for the importance of climate emotions in HE, drawing on our collective experience of the Climate Lab project (2021–2023), a series of in-person and online workshops that brought together scientists, engineers, and artists. We analyse the roles of grief, vulnerability, and creativity in the conversations that occurred, and explore these exchanges as potential pathways out of socially organised climate denial in neoliberal institutions. By drawing on the emerging field of “emotional methodologies,” we make a case for the importance of emotionally reflexive practices for overcoming an institutionalised disconnect between feeling and knowing, especially in Western-disciplinary contexts. We suggest that if staff and students are afforded opportunities to connect with their emotions about the CEC, then institutional transformation is (a) more likely to happen and be meaningfully sustained and (b) less likely to fall into the same problematic patterns of knowledge and action that perpetuate these crises. This profound, sometimes uncomfortable, emotionally reflexive work is situated in the wider context of glimpsing decolonial futures for universities, which is an integral step towards climate and ecological justice.

## 1 Introduction

“*No one talks about climate or ecological crises in my department - not in work time, not at work meetings. Let alone their feelings. It's an extraordinary taboo. I am always thinking about it, yet never feel ‘allowed' to mention it”* (Early Career Researcher, Swansea University).

In 2020, a 12-year-old Japanese pupil researching a class project asked co-author Murray, a glaciologist, a question that she had never been asked in her decades-long, highly successful, scientific career: how do you *feel* about the changes you are seeing at the poles? This question momentarily floored her, and set in train questions of her own, about what would happen if more scientists were asked about their feelings regarding the dire consequences of the climate crisis that they engaged with on a daily basis. Scientists are traditionally expected to view the world through the lens of the scientific method with its requirements for objectivity, repeatability, and logic; they communicate via a precise language of data, graphs, and models. Most climate scientists also undertake public engagement to interpret their knowledge for a general audience and policymakers, but have, for decades, tended to “err on the side of least drama” (Brysse et al., 2013) in their communications—with some notable exceptions (e.g. Carrington, 2024). Within long-standing constraints and expectations, expressing personal thoughts and emotion about the climate (and ecological) crisis is still an extraordinary taboo. And yet, despite all the outstanding and unequivocal science, emissions continue to rise, and the pace of policy and behaviour change is too slow (IPCC, 2023; Stoddard et al., 2021). As Pancost (2022) points out, the failure is not necessarily from lack of trying—some climate scientists have been advocating for action for decades—but efforts are hamstrung by a profoundly conservative and neoliberal research culture that tends to favour only particular (politically-palatable) types of expertise and “advice.” Indeed, if the purpose of universities is to improve society and be agents of change, then it seems that (climate) “science-as-usual” is failing.

Climate Lab emerged from the intersection of our glaciologist's epiphany and a generalised frustration amongst colleagues in other disciplines at Swansea University about the state of Higher Education (HE) and its seeming inability to drive meaningful climate action. It brought together an interdisciplinary team (from social sciences and humanities, biosciences, engineering, and physical geography) with artists and facilitators who had the skills and creative approaches to enable a “deep dive” into participants' climate emotions. After an in-person pilot consisting of two, day-long workshops in 2022, Climate Lab evolved into an online space, with virtual workshops and international participants in 2023. In this article we draw on our experience of creating and participating in Climate Lab to make a case for the importance of emotional methodologies (EMs) that acknowledge the personal, psychological, even spiritual, dimensions of the CEC (Hamilton, 2020) for catalysing and sustaining meaningful action. Here we share our experience of bringing EMs into a university environment, and what might be learnt from this.

**Figure 1:**
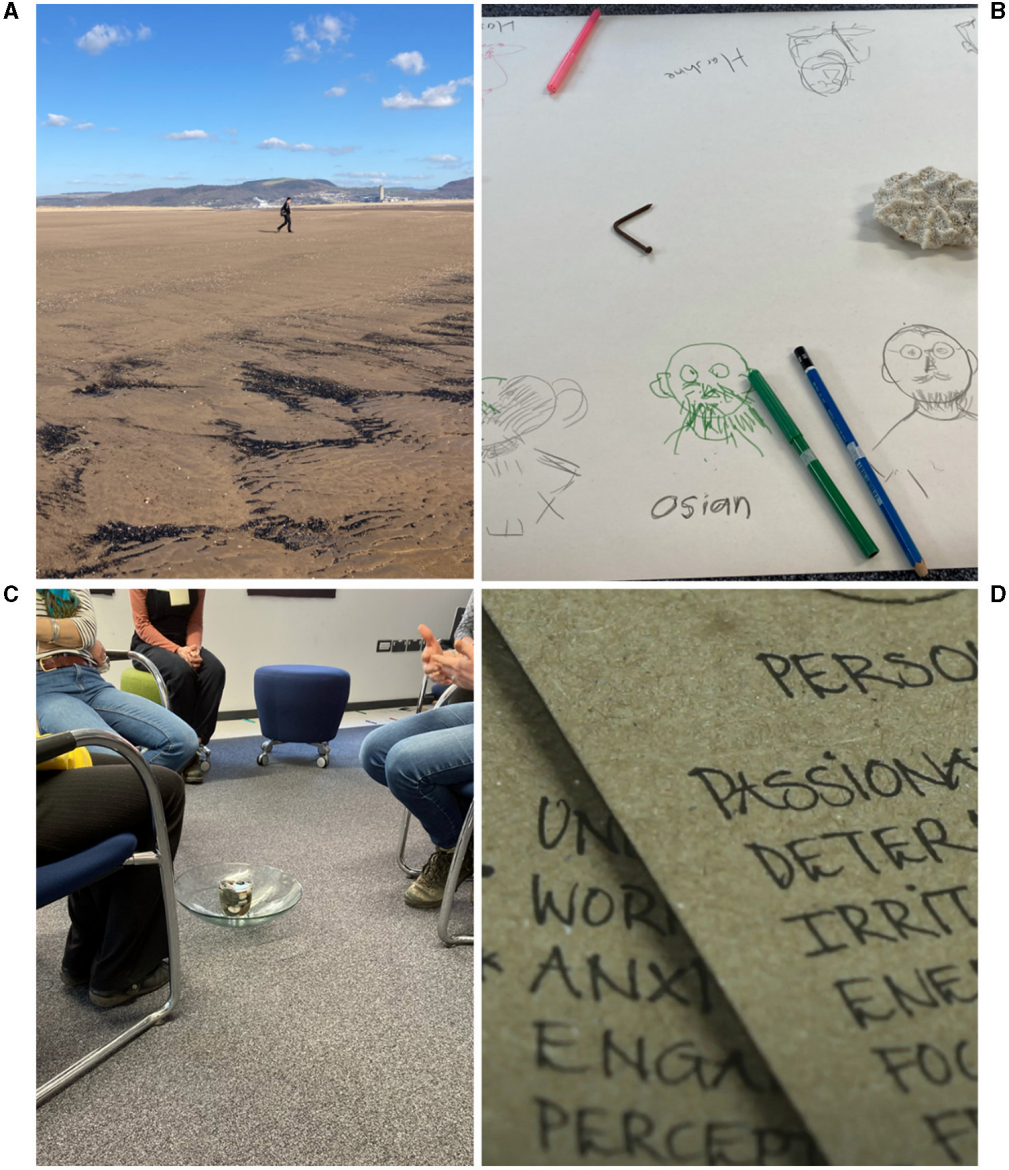
Photos from the Climate Lab pilot: **(A)** Climate lab participant undertaking a ceremonial walk on Swansea Bay beach at low tide; **(B)** drawings of each other made with our non-dominant hands; **(C)** the “fishbowl” Council method, whereby a few participants sit in the centre of a circle to talk, while those in the outer circle listen (some pebbles and sea water in a glass bowl placed in the middle add to the sense of ritual); **(D)** some of the participants' descriptions of their personalities and emotions, recorded by Marega Palser.

We first contextualise Climate Lab with an overview of the current predicament of universities (focusing on the UK where we are based) and how they (and we, as academics) might be made fit for purpose in an era of escalating climate and ecological crises. We then discuss the emerging field of emotional methodologies and their relevance for overcoming socially organised denial and climate action inertia in HE, before describing Climate Lab's format and content, and briefly discussing the importance of art and creativity in the process. In the findings and discussion, we draw on our own, other participants', and the artists' responses to the workshops (recorded in post-workshop feedback via Google Docs, and completed by 10 participants), as well as observations made by co-author Pigott during the workshops and in subsequent discussions about Climate Lab with team members, to explore the key themes that emerge from the Climate Lab process (Figure 1) in relation to wider literature. We consider how Climate Lab is a particularly distinctive and useful approach in a university setting: the sustained engagement with emotions that helps process disenfranchised grief, and the potential of emotional methodologies for catalysing personal and collective agency. We conclude the discussion section with some challenges faced in terms of our responsibilities, in a predominantly white and western context, to decolonise our universities.

As Climate Lab was primarily intended as an experimental space for changing ways of working in the university, and funding did not cover a research budget, no formal methodology or analytical framework is applied beyond a broadly thematic analysis of various material and sources of data that arose from the workshops. Rather, our preliminary reflections on Climate Lab in this paper are intended to open up conversations on this topic and demonstrate the value of further research, funding and action.

## 2 Contextualising Climate Lab

### 2.1 Why universities, why now?

Universities have taken a leading role in generating knowledge about the climate crisis over the last several decades, and although almost every government in the world acknowledges and pledges action to address climate change, the emissions curve trends ever-upwards (Stoddard et al., 2021). Given the scale of societal transformation needed, universities could be pivotal change agents (Giesenbauer and Müller-Christ, 2020). However, despite thousands of higher education institutes declaring climate emergencies, it seems that they are poorly-equipped to fulfil their responsibilities as part of societies' critical learning infrastructure and contributors to public good (Facer, 2021; Gardner et al., 2021; Green, 2021). While some argue that this demonstrates that the science-society contract is broken and in need of reformulation (Glavovic et al., 2021), others go as far as accusing universities of betraying humanity (e.g. Green, 2020; Maxwell, 2021) and becoming “fraud bubbles” on account of the double reality that staff and students must live and construct in order to function in an environment that is maladaptive to taking the CEC seriously (Thierry et al., 2023).

There is clearly more that universities could do. A recent statement from the Independent Social Research Foundation notes that the current crises are “deepened by a knowledge crisis. Not enough research is funded, or is not of the right kind, or is not properly integrated across cultural, economic and scientific fields, or is ignored by the public, or refused by governments, or denied by industry, or distorted by the media. Many of us have become fatalistic about these problems in a time when research needs to address them.”^1^ Others argue that tweaks to research agendas are not enough, given the scale and urgency of the emergency. A slew of recent papers urges academics to step outside their research roles and ramp up their public advocacy, peaceful civil disobedience, and even issue moratoriums on climate science until politicians heed its advice (Gardner et al., 2021; Capstick et al., 2022; Racimo et al., 2022; Glavovic et al., 2021). These commentators argue that all these tactics are justifiable given the severity of our planetary circumstances and because academics have a moral responsibility to act in ways that are commensurate with what they know (Thierry et al., 2023). In short, academics are beginning to engage in the (climate) politics and values that most—especially in Western-scientific contexts—have been trained to put to one side in the interest of scientific neutrality, impartiality, and integrity (Green, 2020; Head and Harada, 2017). This imperative intersects with and is part of wider moves towards decolonising universities (Smith, 2021, Bhambra et al., 2020; Radcliffe, 2017), that are centred around responsibility and accountability, listening and reciprocity. Decolonising practices reject the highly colonial image of the scientist/researcher as a detached observer, and instead argue that research/researchers ought to speak truth to power and become allies of the groups and causes with whom they work (Radcliffe, 2017).

The climate crisis (and intersecting ecological, racial, and inequality crises (e.g. Sultana, 2022)) therefore demands a wholesale reimagining of HE and what it should do (Facer, 2021; McGeown and Barry, 2023). Suggestions of what a climate-serious university might look like range from providing training in community engagement, advocacy, and media communication, to providing staff with security to engage in civil disobedience, and using campuses as hubs for community organising (e.g. Gardner et al., 2021). In her report, “Beyond Business as usual: Higher education in the era of climate change” Facer (2021) sums up the changes required in terms of four overarching themes: “(i) Redesigning the day-to-day operations of universities and colleges to reduce emissions, nurture biodiversity and adapt to the impacts of a changing climate; (ii) Reinvigorating the civic role of institutions to build ecologically and socially resilient communities; (iii) Reshaping the knowledge structures of the university to address the interdisciplinary complexity of climate change; and (iv) Refocusing the educational mission of the institution to support students [and we would add, staff] to develop the *emotional*, intellectual and practical capabilities to live well with each other and with the planet” (Facer, 2021, p. 6; emphasis added). Such propositions and visions for the future of HE are exciting, and we support them wholeheartedly, although we recognise that they will not be easy. Given that the question of *how* these visions can be achieved is often overlooked (Owens et al., 2023; Card and Closson, 2023), our focus on emotions in this paper is intended to strengthen the movement and increase the likelihood of such visions becoming reality.

### 2.2 Why emotions?

“*Information is not changing our minds*—*most people make decisions on the basis of feelings” (Eno*, *2022**)*.

It would be surprising if our current planetary predicament did *not* generate emotional responses (Head and Harada, 2017), yet recognition of the emotional dimensions of these crises has only recently begun to gain traction and is still limited in environments heavily invested in scientific knowledge (such as universities). Anderson and Smith (2001, p. 7) argue that the neglect of emotions leaves a “void in how to both know, and intervene in, the world,” and that this gendered production of knowledge side-lines emotions, favouring a (masculinised) “detachment, objectivity, and rationality” over a feminised “subjectivity, passion and desire”.

Although “affect,” “emotion” and “feeling” can all be variously defined, for the purposes of this paper we approach these concepts interchangeably, to denote a cocktail of unconscious bodily feelings and conscious experiences of feelings (Hamilton, 2020; Pihkala, 2022). Indeed, the climatic and the affective are entangled; as Verlie attests, climate change—as a phenomenon that is *felt* through things like temperatures, hurricanes, disease, floods, and drought—“reconfigures, disrupts, shapes and directs humans, and everyday human affective practices contribute to changing or stabilising climate” (Verlie, 2019, unpaginated). Once we acknowledge the interplay of emotions in everything we think and do about the climate crisis, we can acknowledge the social, political and cultural context of the emotions we feel, how we manage them and how this influences the kinds of actions we do or don't take (Ahmed, 2014).

A raft of past research demonstrates the importance of emotions both for understanding the climate crisis and for responding to it. Norgaard (2006) found that people manage their emotions in line with social norms (effectively suppressing distressing emotions), producing a kind of “everyday denial,” and subsequent research has investigated the emotions associated with the climate and nature crises (Cunsolo Willox et al., 2013; Duggan et al., 2021; Wang et al., 2018; Hickman et al., 2021), created new vocabulary (Albrecht, 2019), and explored the relationship between emotions and environmental actions (Norgaard, 2011; Sangervo et al., 2022). Davidson and Kecinski (2022) suggest that understanding emotions is critical to the success of adaptation and mitigation strategies. Increasingly, research highlights that distress and anxiety are emotions particularly associated with the climate crisis, including studies with children and young people (Hickman et al., 2021), the general public (Sangervo et al., 2022; Whitmarsh and O'Neill, 2011), affected communities (Tschakert et al., 2013; Askland and Bunn, 2018), educators (Verlie et al., 2020), and climate scientists (Head and Harada, 2017; Duggan et al., 2021). Rather than unfairly shifting the burden of responsibility for climate action onto individuals (instead of the wider political and institutional drivers of the CEC), such research underpins the assertion made by Ahmed (2014) and Verlie (2019) (see above) that emotions cannot be considered separately from the social-political realm; the capacity for changes in one is dependent on the other, and *vice versa*.

The subject of this paper and the project—Climate Lab—is not so much concerned with *which* emotions are connected to climate action, but rather how creating a space to express and process *any* emotion about the crisis can itself open up new possibilities for personal and collective transformation. From a psychological perspective, it has long been observed that managing emotion involves “bodily preparation for a consciously or unconsciously anticipated deed” and that “this is why emotion work is *work*, and why estrangement from emotion is estrangement *from* something of importance and weight” (Hochschild, 1983, p. 230). This is also true of the CEC, where suppressing emotion is more likely to result in poor mental health, isolation, inaction and apathy (Norgaard, 2011; Lertzman, 2015; Gordon et al., 2019) than it is to result in meaningful progress. And so, while the pursuit of objective, rational science will remain an important part of university climate research, it can simultaneously estrange staff from their feelings about the climate and ecological crisis. Neglecting these feelings, we suggest, may prevent Higher Education Institutes from becoming the agents of social change that they could—and many would argue, *should*—be.

### 2.3 Emotions in a university setting

Studies have shown how scientists enlist particular behaviours, strategies and energies to keep their emotions hidden and maintain their image as objective and rational (Head and Harada, 2017; Gillespie, 2020). Such management can be thought of as a form of emotional labour (Hochschild, 1979, 1983), which as we discuss below, has inner (wellbeing and mental health) and outer (ability to effect or participate in change) implications.

A doctrine of impartiality and objectivity in academia—and especially in science disciplines—has created various social “defences” (Randall and Hoggett, 2019) to keep climate emotions at bay. Indeed, Brysse et al. (2013) found that “dispassionate norms” creates a bias towards toning-down statements that might be perceived as alarmist. Furthermore, where emotions about uncertain environmental futures are expressed, they tend to emphasise resilience, adaptation, and risk management which are—arguably—more positive emotional outlooks (Rickards et al., 2014). Research by Head and Harada (2017) with climate scientists in Australia found several common triggers for downplaying emotions: (1) the social norms of science (rationality); (2) fear of attacks from climate denialists; (3) personal denial in order to protect self and family in everyday life; (4) maintaining an optimistic disposition in order to maintain personal and group resolve (to do science); and similarly (5) a focus on pleasurable emotions about their jobs (i.e. it being interesting and fun work) which galvanised a sense of scientific community and fellowship. Consequently, Thierry et al. (2023, p. 2) note how “...on a day-to-day basis, most academic staff seem to be maintaining the semblance of normalcy and unconcern. So great is our apparent collective indifference that an onlooker could be forgiven for thinking that we do not believe our own institutions' official warnings that an emergency is unfolding around us.” They identify organisational structures of modern higher education institutes that uphold an extractivist growth economy and legitimate hegemonic cultural practices as a primary cause of inbuilt inertia. Such inertia, coupled with a psychological need to deny the consequences of our own inaction, makes it very difficult for individuals within the organisation to challenge the status quo (Thierry et al., 2023). Thus, climate silence is a blockage along the pathway(s) towards genuine transformation.

However, emotional restraint about the climate crisis amongst scientists may be reaching a breaking point, with many increasingly compelled to voice their feelings (e.g. Harrabin, 2022; Gardner and Wordley, 2019; Green, 2020). Academics are increasingly speaking out about the emotional toll of their climate knowledge: in a short letter to the journal, Science, titled “Grieving environmental scientists need support”, Gordon et al. (2019) note how the losses associated with the climate and ecological crises trigger strong grief responses amongst people with an emotional attachment to nature, but that “environmental scientists are presented with few opportunities to address this grief professionally” (Gordon et al., 2019, p. 193). Pihkala (2020) also suggests that provision and support for academic staff to process their eco-anxiety will be essential for personal growth and transformation, and therefore also for the work of putting higher education on a path towards climate justice. Again, such research demonstrates how individual and institutional changes are fundamentally intertwined and thus how genuine transformation needs to include both—rather than unfairly directing blame or responsibility on one or the other. Our approach, exemplified through Climate Lab, offers a way in which to do this by providing space and time to acknowledge, share, and process hitherto neglected emotions about the CEC.

## 3 Methods

### 3.1 Emotional methodologies

Despite mounting evidence (and indeed, ancient wisdom and common sense) that inner dimensions (such as emotions, affects, value systems, and mindsets) are fundamental to how people engage the world and respond to problems, climate and ecological crises are still primarily approached as external problems to be addressed through “outer” changes in science, technology, and politics. Such neglect is likely hindering any efforts towards meaningful and deep transformations for more liveable futures (Ives et al., 2020). External factors may be the *least* likely place to produce sustained change if inner dimensions are not also addressed; many studies, from a host of disciplines, now show that emotions and mindsets can be “deep leverage points” for transformation at individual, cultural, and political scales (Hamilton, 2022; see also Meadows, 1999; O'Brien, 2018; Wamsler et al., 2020; Woiwode et al., 2021; McCaffrey and Boucher, 2022). Davidson and Kecinski (2022, p. 1) go as far as to say that “the first trigger to any personal and collective change begins with emotions…Emotions are thus at the centre of social responses to climate change.” Although a strict binary or boundary between internal and external is untenable (see Ahmed, 2014), it is helpful to think of the overlap of what O'Brien (2018) calls the “practical, political and personal spheres” of transformation, and how they influence one another. In neglecting the personal sphere, we neglect a significant—perhaps even dominant—sphere of influence (see also O'Brien, 2021).

“Emotional methodologies” (EM) are a way to acknowledge, explore, and encourage the processing of complex emotions in a safe and contained way. Key to EMs is the development of emotional reflexivity, defined as “an embodied and relational awareness of—and attention to—the ways that people engage with and feel about issues, how this influences their responses, the actions they take, the stories and worldviews they inhabit and their perceptions of individual and collective agency” (Hamilton, 2022, p. 4. See also Pain, 2009; Holmes, 2015). Developing emotional reflexivity is influenced by the “emotional habitus”; that is, the “safe spaces” in which to acknowledge and explore emotions, and the presence or absence of social norms that denote particular ways of emoting that either avoid or welcome uncomfortable emotions about climate (Gould, 2009; Norgaard, 2011; Owen et al., 2022; Hamilton, 2022). At their core, emotional methodologies challenge what we mean by “communication” in the context of CEC. Despite all our sophisticated options for communication, we might ask whether the deepest purpose of communication, to create understanding and foster connection and, ultimately, to ensure survival, is being served? As Moser (2015) suggests, given the dire straits we find ourselves in, perhaps not. She argues that what is needed most is not persuasion, education, and deliberation (the hallmarks of climate education and communication within HE and beyond), but rather kindness and compassion, respect, and dignity: “Not a battle of the minds, but a meeting of the hearts” (ibid.).

These can be tough words to absorb in a culture built on ways of knowing that prioritise rational thought, debate, and impartiality. The traditional university, and particularly the neoliberal one (Thierry et al., 2023) does not make a natural emotional habitus because it side-lines, even suppresses, many other ways of knowing—bodily, emotional, spiritual, intuitive—that we know are central to how people come to understand and respond to environmental change. And while a range of approaches do increasingly see researchers as subjective, active participants in knowledge creation, and there is a rich tradition of centering emotion and affect in some disciplines, these endeavours do not currently hold authority on climate change, both in terms of who convenes research initiatives in universities, and who communicates about the crisis to the public (Gardner et al., 2021). Making room for embodied and emotional knowledge is also an important part of decentering and disrupting the imperial, Western knowledge systems that are intimately bound up with colonialism and climate (Smith, 2021; Sultana, 2022). Other research shows how emotions and emotional methodologies are implicated in long term individual and collective resistance in autonomous forms of activism (Brown and Pickerill, 2009; Jasper, 2011). However, only a small body of work has investigated the emotional landscapes of universities and education settings in relation to the climate crisis (e.g. Willis, 2012; Head and Harada, 2017; Jovarauskaite and Böhm, 2020; Jones and Davison, 2021; Verlie et al., 2020), and still fewer^2^ propose methods for overcoming the anxiety, avoidance, and inertia (amongst staff and students) that many of these studies observe.

### 3.2 Climate Lab

As noted in the introduction, the catalyst for Climate Lab was a child's question to glaciologist Murray regarding her feelings about the climate crisis. Murray subsequently brought together an interdisciplinary team of academics at Swansea University to explore what would happen if other scientists were asked the same question. It became evident that we would need skilled facilitators to create the kind of space where academics would feel comfortable to discuss their feelings. For this, we turned to an organisation called Emergence (https://emergence-uk.org/about/), based in mid-Wales, with whom one member of the team had interacted before (Pigott, 2020). Some university seed corn funding enabled us to commission Emergence, and the Climate Lab pilot project was born.

The Climate Lab pilot consisted of two, day-long, in-person workshops in March 2022, at two locations at Swansea University's campuses, both facilitated by artist and founder of Emergence, Fern Smith,^3^ and Newport-based performance artist, Marega Palser.^4^ Two other artists from south Wales, Emily Hinshewood and Tanya Syed, were commissioned to join the workshops and produce creative responses. Invites were circulated within the Faculty of Science and Engineering at Swansea University and aimed at “climate scientists and engineers.” We focused on STEM disciplines because these disciplines embody a culture of science that most strongly denies or hides the emotional dimensions of doing science (Willis, 2012; Brysse et al., 2013), and such suppression is increasingly understood to be a barrier to effective action (Brown and Pickerill, 2009; Head and Harada, 2017; Randall and Hoggett, 2019).

The invite called people “to participate in a unique, immersive, experiential research lab focusing on climate change, sea level rise, and the future coastline of Wales.” It asked questions such as “Can we take the expert viewpoint of climate scientists ‘outside of the box' of the scientific method?”' and explained that the intention would be to “step into a new space for enquiry,” “share and listen to stories from others involved in climate research,” “see your work from a fresh perspective,” “examine and witness the impact of climate research on those who undertake it,” and “engage in mutual inspiration and co-learning” with an “emphasis on creating an atmosphere of trust and reflection—providing space and time for emotions to be shared.” The pilots involved 16 participants (including the organising team and facilitators) from Engineering, Geography, Biosciences, and Health and Human Sciences, ranging from postdoctoral researchers to professors. Most, but not all, were white, and only three participants were men. Although no respondents to the invitation were turned away, the number of participants was around the upper limit that the facilitators had deemed optimal for an in-person workshop.

To create an atmosphere of trust and reflection during the first workshop, the facilitators steered our energies into activities that would help us get to know one another and to feel at home in the space. Some were “fun” activities such as simple body and breath work and drawing pictures of one another with our non-dominant hand without looking at the page (see Figure 1). In another activity we were asked to choose an object from an array of trinkets, found objects, flotsam and jetsam laid out on a table, and to share with another person how our chosen object resonated with us. We were asked to reflect on our personalities, values, and deepest questions. These activities were surprising and perhaps uncomfortable for some, particularly as there was little in these first activities that had anything obviously to do with the climate crisis. A couple of participants dropped out between the first and second workshops.

The first part of the pilot Climate Lab, held in a building a stone's throw from the beach, incorporated ceremonial aspects such as walking out in silence at low tide to meet the sea (a long walk at Swansea Bay, where the tidal range is 8 m), and the use of a talking circle known as “council.” Council circles use a very specific way of speaking in turn, from the heart, with specific guidelines for sharing (Zimmerman and Coyle, 1996). This means talking only about personal feelings rather than jumping to solutions and offering what one thinks ought to be done about a particular situation; this is a subtle but important distinction in the climate context where discussions often revolve around solutions and blame (Pigott, 2020). The invitation to speak in circle was in response to the phrase “knowing what I know and doing what I do, my greatest fear for the future is….” Other participants were encouraged to actively and deeply listen (rather than respond, debate or plan what they wanted to say next). This enabled a form of witnessing (Macy and Brown, 2015) that not only helps people to feel more in touch with what they feel themselves, but also helps them understand that others may feel the same; this can be a galvanising experience (Johnstone, 2002; Pigott, 2020).

**Figure 2:**
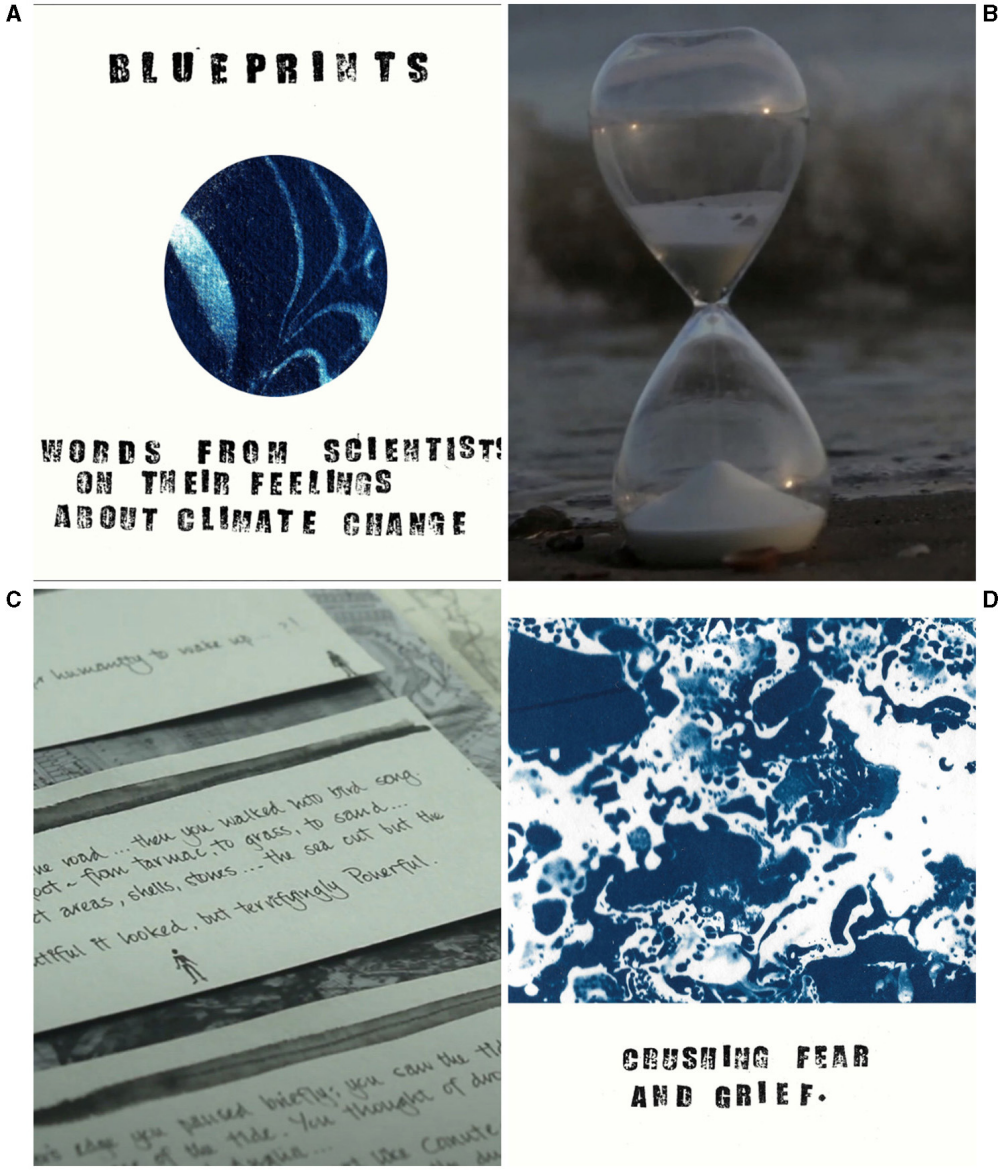
Snapshots of the artists' creative responses/works in progress during the pilot Climate Lab: **(A)** “Blueprints” by Emily Hinshelwood, a series of cyanotype postcards featuring participant's words in response to the prompt “knowing what I know and doing what I do, my greatest fear for the future is…”; **(B)** A still from “Islands of Possibility”, a film by Tanya Syed, that explores the role of time in emotions about the CEC (see https://vimeo.com/737337900); **(C)** Participants' words scribed and arranged around a map of Swansea Bay, which were spoken and laid out in a ceremonial fashion during a performance piece by Marega Palser; **(D)** One of the postcards by Emily Hinshelwood.

During part two of the pilot three weeks later, the same participants came together to experience the artists' creative responses^5^ (Figure 2), and to hear about their creative processes in response to what they had experienced during the first workshop. Further creative, ritualistic and ceremonial practices were employed, including short walks, talking circles, a tea ceremony, and drawing/writing.

The workshop methodology, although somewhat alien in a university setting, was inspired by a framework for transformation—Joanna Macy's Work that Reconnects (WTR)—that is widely used elsewhere, particularly in activist and community spaces. The WTR is a loose framework that was developed by Macy and colleagues in the 1970s (Macy and Johnstone, 2012; Macy and Brown, 2015) and continues to evolve. It draws on a combination of systems theory, Buddhist philosophy and deep ecology and has at its core the aim to connect people to their emotions, to others and to the more-than-human world. Research into the impacts of WTR by practitioners has found that it can strengthen connections to self, others, and the more-than-human world, and that workshops can renew commitment to action (Johnstone, 2002; Hollis-Walker, 2012; Hathaway, 2017). Climate Lab followed the four-part structure of the WTR. On the first day the participants were led through the “coming from gratitude” and “honouring our pain for the world” stages, and on day two the focus changed to the “seeing with new eyes” and “going forth” stages.

After the pilot in 2022, Climate Lab secured further internal funding to run two online iterations of the workshops in 2023; both were two-part processes, Global Climate Lab 1 in May and June, and Global Climate Lab 2 in September and October. This time, invites were sent out internationally via email, twitter, LinkedIn, fliers at conferences, and departmental newsletters, aimed at “climate researchers” (after receiving interest from social scientists, we decided to broaden the focus from STEM-only disciplines). The first workshop received 11 participants from the USA, Canada, Denmark, Germany, Pakistan, and the UK. The second workshop had 15 participants from Australia, France, Pakistan, Bangladesh, Germany and the UK. Most participants were female (four participants were male; of these, only one attended both parts of their Lab). Again, no respondents to the invite were turned away unless they knew in advance that they would not be able to attend both parts of the workshop. Facilitators planned activities for up to 15 online participants, although larger groups could potentially be accommodated by adjusting the methods.

A call was put out via Emergence's networks for an artist to participate in each workshop. From a number of applicants, two were commissioned—multidisciplinary artist Carolina Caycedo (based in the USA), for the first workshop, and Christine Kettaneh, a sculptural and performance artist (based in Lebanon), who joined the second workshop.

These online workshops followed the WTR framework in a similar way to the pilot project, with activities adapted by our facilitators to work in an online environment (Zoom). A third and final online “celebratory gathering” was offered for each cohort to showcase the outcomes of the artists' endeavours (these were shown as “works in progress” during the second workshops but finalised by the third gathering). The Council method was, again, a critically important component of the online Climate Labs, and the “hide/show/pin” video functions in Zoom helped to create a virtual space that facilitated focus and deep listening to whoever was speaking.

The ethics process was made more robust for the Global Climate Labs after it was flagged to us during the pilot that the invitation to bring emotions to the fore could be triggering for some. In addition to seeking the usual university ethics clearance, we also asked participants to read detailed information about the workshops and sign a consent form, and we built in more “support” spaces, including a breakout room option for anyone needing someone (a nominated team member) to talk to, and by signposting various support services related to climate distress.^6^ Each workshop began by stressing to participants that the nature of the climate and ecological crises meant that there could be no guarantee of an entirely “safe space”; indeed, the aim was to create “safe enough” or “brave” (Arao and Clemens, 2023) spaces in which participants felt able to encounter their (and others') most uncomfortable and upsetting feelings about the crises.

### 3.3 Creative participation

The invitation to engage in mutual creativity alongside professional artists was at the heart of Climate Lab and enabled it to create space for emotions and connections that are otherwise difficult to access. From the start of the planning process, our facilitators were clear that they would not simply be facilitating discussions *between* climate scientists and engineers whilst artists merely observed and “reported back” on the process. Similarly, we (the organising team) were strongly encouraged to participate rather than observe as “researchers.” This approach resonates with participatory action research (PAR) as well as decolonial and feminist approaches to knowledge co-creation, whereby all participants' various knowledge(s) and expertise are valued equally (see, for example, Omodan and Dastile, 2023; Country et al., 2016; Smith, 2021; Haraway, 2016). Importantly, such approaches reject a notion that researchers are objective bystanders to the worlds they research, because we are all always and unavoidably part of the world, influencing how events unfold and how knowledge is created (Barad, 2007; Ingold, 2016). PAR is also primarily focused on creating societal *change*, rather than simply “data” (Kemmis, 2010). As co-author and facilitator Smith elaborates in a series of blog posts^7^ about Climate Lab,

“…although easier to distinguish and separate roles from a research point of view, this would set up a false division between ‘us' and ‘them' - one often replicated in projects which invite artists into scientific forums. This risks the artists becoming instrumental and secondary to the scientists, rather than both learning from each other, shaping the narrative, and creating change together.”

Creative methods and the involvement of artists were central to Climate Lab's transformative potential because they created a conducive “emotional habitus” (Hamilton, 2022) for the sharing and processing of emotions, giving participants opportunities and permission to access different (often more playful, imaginative, or deeper) parts of themselves and different ways of interacting with one another than they are usually accustomed to in an institutional setting (these themes are explored more fully in Pigott et al., in preparation).

**Figure 3:**
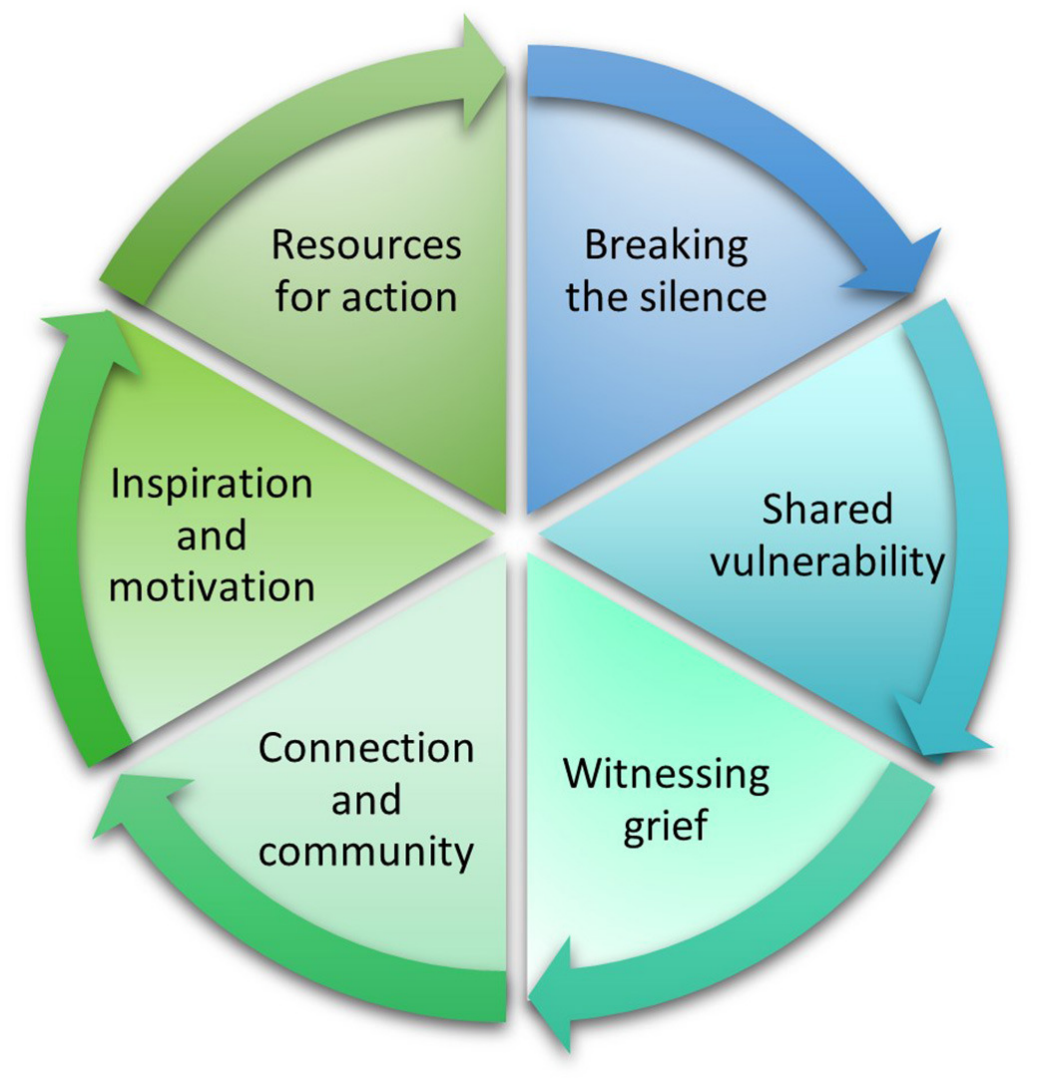
Conceptualised outcomes of Climate Lab, loosely based on the Work That Reconnects framework (Macy and Johnstone, 2012; Macy and Brown, 2015).

In what follows we describe and discuss the various effects of, and themes arising from, the pilot and Global Climate Labs (conceptualised in Figure 3) in relation to their potential to catalyse transformation in HE.

## 4 Findings and discussion

### 4.1 Breaking the silence

“*The power of grieving connects us…we discover that others feel the same way as we do*—*even in a university divided by campuses, disciplines, and departments. We find that we are not alone. Grief makes us reach out for support. It creates a community; it has the potential to create a village within an institution. This galvanises us and makes us more resilient. It makes us attend to what is important and helps us keep on keeping on.”* Fern Smith, Climate Lab creator and facilitator (see text footnote^7^).

**Figure 4:**
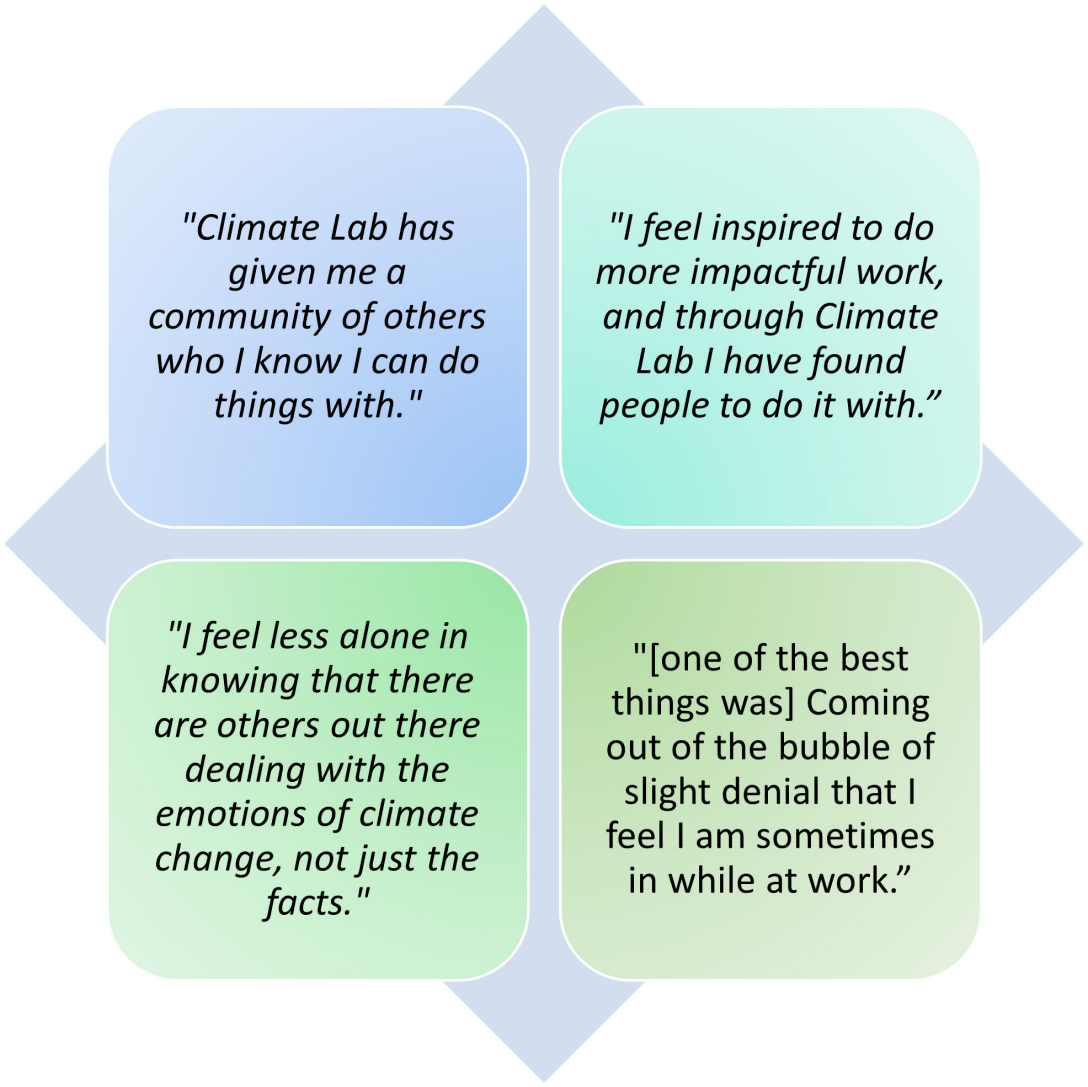
Climate Lab participant feedback, collected via a post-workshop online survey.

In a very direct sense, Climate Lab provided spaces of connection that helped break a generalised climate silence in HE and “burst the bubble” of pluralistic ignorance whereby individuals hold a false assumption that no one else cares (Geiger and Swim, 2016; Thierry et al., 2023). Participant comments indicated that hearing how their colleagues also cared about the climate was a welcome revelation (Figure 4). These moments of interpersonal connection can spark processes of social contagion within and beyond institutions (Thierry et al., 2023; Moser and Dilling, 2007; Winkelmann et al., 2022), as people gather confidence that their views and values are shared by others. From a systems change perspective, when a system (i.e. the individuals comprising that system) has the opportunity to “see itself,” then it gains power to imaginatively transcend that system/paradigm and—in the language of the Work That Reconnects—to “see the world with new eyes” (Macy and Brown, 2015). The value of such a rupture in the daily fabric of how life is imagined, of the daily illusion that no one else cares and that norms and cultures are unquestionable, cannot be underestimated because it represents a significant leverage point for exerting change in social systems more widely (Meadows, 1999).

Spaces of connection and community-formation (whether brief or ongoing) can give people the courage, camaraderie and “deep determination” to take climate action (Hamilton, 2022). Speaking about the CEC can be difficult, especially in work settings, but is vital. Although the Climate Lab organising team already knew each other in some capacity, the bonds we forged with one another and with other participants during the in-person pilot Climate Lab (and the more ephemeral but nonetheless powerful connections made with international researchers during the Global Climate Labs) have been of a different quality to that of day-to-day collegiality. Having seen one another express deep concern and vulnerability about the CEC (including, at times, tears), we have subsequently found ourselves encouraged and emboldened to bring up the CEC as often as possible in our workplace and lives (see section “Pathways towards personal and collective agency,” below), from university committees to grant review panels, and through public outreach and engagement activities. Without adequate emotional support networks, doing so is an immense pressure and responsibility—that requires considerable bravery—for climate-concerned HE staff who are already likely to be overworked, precariously employed, and feeling isolated by the culture(s) they operate within (Owens et al., 2023).

Sharing distressing emotions makes us vulnerable, and although uncomfortable, such vulnerability presents a way of (re)connecting with one another, bringing to the fore a subjectivity that is—crucially—*receptive*; we become better able to think and feel our interdependence with one another, and also our corporeal vulnerability to and dependence on the more-than-human world (Butler et al., 2016; Verlie, 2021). Rather than understanding vulnerability only in material and political terms (i.e., “climate vulnerability”—an approach which tends to imaginatively set people apart at global and local scales), Eriksen helpfully suggests that “vulnerability is fundamental to the connectedness in social relations critical to understanding and acting on climate change” (Eriksen, 2022, p. 1279), and urges us to investigate the deeply personal realms of vulnerability that relate to linking lived experiences and a shared humanity (Eriksen, 2022).

Leaning into vulnerability also means refusing to turn away from the intractable contradictions and difficulties of these times, which can otherwise side-line the kinds of knowledges, subjectivities, and practices that are better able to cope and thrive with complexity and difference (Pigott, 2020); As Solnit proffers, within the spaciousness of uncertainty there is room to act (Solnit, 2016; see also Mouffe, 2000). Making space for vulnerability and related emotions such as shame, guilt, and uncertainty is also part of the work of decolonising the (predominantly white, masculine, linear, progress-oriented) knowledge structures that contribute to individualism and environmental destruction (Singh, 2018; Chakrabarty, 2000). In this sense, vulnerability is not an obstacle to climate action, but rather can be a means for generating different kinds of (much-needed) ethico-political awareness, and more communal ways of perceiving and being together (Eriksen, 2022; Ramsden, 2016).

### 4.2 Forming community

Through Climate Lab we experienced ourselves and observed in others how turning towards and expressing difficult emotions enabled a changed relationship with other participants in the group. Participants commented (Figure 4) on having found a community of others in their institution who felt the same as them, and how this gave them inspiration and courage to continue their work and/or take bolder steps. Their comments align with wider research on emotional methodologies, where the “disenfranchised grief” that participants express and share with others can become a resource for initiating and sustaining action (Hamilton, 2020; Verlie, 2019; Randall, 2009; Lertzman, 2015; Head, 2016; Cunsolo and Ellis, 2018; Osborne, 2018).

Recent research indicates that one of the biggest barriers to HE educators taking action is not a lack of access to information, materials or resources but rather the social, cultural and institutional factors which shape educator's agency and opportunities to enact change (Owens et al., 2023). These include organisational culture and epistemic norms, the “tone” set by senior management regarding whether the CEC are taken seriously, academics perceiving risks to career and credibility for appearing “radical” or “political,” an intense institutional focus on efficiency and productivity at the cost of time and space to develop novel approaches to the CEC in universities, and the vested interests of fossil fuel companies in universities which create a conflict of interest for senior management and some staff. The research found that one of the key challenges that educators face is dealing with their own distressing emotions about the CEC (echoing commentaries by Gordon et al., 2019 and Pihkala, 2020) and that day-to-day routine interactions and connections between colleagues in HE are important in capacity-building to enable people to transform their good will and concern into action (Owens et al., 2023).

What the Climate Lab showed us, however, is that processing distressing emotions is difficult and skilled work, and unlikely to be facilitated through “routine interactions” alone; indeed, we know that overall, cultural norms dictate what is kept in and out of discussion in routine institutional interactions—hence the significantly different approach and creative methods used in Climate Lab to enable participants to step out of what was usually expected of them in their roles. Drawing on and extending Owens et al. (2023) work, we therefore suggest that making space for emotions is itself a key conversion factor in determining the capabilities of HE staff to enact change, because—in our experience—bearing witness to one another's emotions established bonds between participants that would not have otherwise existed.^8^ The artists' creative responses were a key part of this witnessing process, reflecting back to the participants the emotions that had been shared, and reinforcing the fact that they existed and had been heard. For example, participants commented that “…it's always enlightening to work with more voices with different lived experiences. I enjoyed seeing and hearing my and others' words/images reflected back and interpreted through the artists' works” and “It was a very good process. I felt so much more of everything.”

The climate crisis is often conceptualised as a crisis of imagination (e.g. Wapner and Elver, 2016), but it is also a crisis of connection (Hodgetts, 2023). Our warming climate is both a symptom and a cause of a centuries-long decline in social connection and community cohesion (Card and Closson, 2023); our increasingly individualised lifestyles, particularly in high-income countries, take a huge toll on the planet (Moon et al., 2023), while increasing temperatures are also likely to further fracture and stress our relationships with one another and increase feelings of anxiety and distress (Card et al., 2023). What is more, research shows that attempting to reverse this decline and to foster social cohesiveness is more likely to be achieved through intimacy rather than information. An experiment by van Swol et al. (2021) showed that when discussion groups were encouraged to engage in self-disclosure and focus on shared values, they had higher ratings of social cohesion, group attraction, and collective engagement (and lower ratings of ostracism) than those groups encouraged to solely discuss information from an article about climate change.

This observation gives us clues as to why Climate Lab felt like such a radical space within the university and even as an online space. It affirms something that is well known in feminist and post-colonial research, but less examined in discussions about the CEC, or indeed HE, which is that emotions are political, they enact change in the world, and facilitate the formation of communities and movements (Ahmed, 2014). As Verlie insists, the work of caring and of building caring communities is a form of climate action; “emotional work is political work” (Verlie interviewed by O'Neill, 2022). In fact, this work of building caring communities might be one of the most promising edges of climate action: leading Intergovernmental Panel on Climate Change scientists such as Christina Figures and Karen O'Brien are increasingly turning to examine the inner and collective dimensions of experience that underpin climate action inertia—and might be key to overcoming it (Green, 2022; Bristow et al., 2022; O'Brien, 2021). This includes approaching the CEC itself as a “collective trauma” of mass numbing, denial, and avoidance of responsibility by leaders and wealthy nations for the traumas of colonialism and climate change for which they are primarily responsible (Green, 2022).

Approaching the CEC as a trauma legitimises using collective healing practices and “radical tenderness” (Machado de Oliveira, 2021, p. xxi) to recognise feelings of individual and collective helplessness, shame, fear, and grief and to tap into the wisdom this may reveal (Green, 2022). Such feelings were welcomed in Climate Lab; one participant commented that they appreciated having the opportunity to sense “the urgency of what is causing these feelings of doom in scientists. It was raw… a more contained emotion, one almost laced with guilt.” Although Climate Lab never explicitly used the terms “trauma” or “healing” both facilitators having training and experience in working with distress and trauma. The design, activities, and presentation of Climate Lab was thus informed by trauma healing approaches, such as multi-day processes of trust-building, slowing down, communicating precisely, attuning to others, and recognising unacknowledged emotions—and in doing so aiming to build more collaborative (and capable) communities (Green, 2022).

### 4.3 Pathways towards personal and collective agency

Perhaps the most telling observation is that of our own (the organising team's) experiences of how Climate Lab changed our relationships with one another and our capacities to make change in our institution. Although we all knew each other professionally prior to Climate Lab, it is not an exaggeration to say that these relationships have been considerably deepened through the experience of hearing one another speak so openly, emotionally, and vulnerably about the CEC. These connections have paved the way for actions that we have subsequently each gone out into the university to initiate or participate in (both individually and in collaboration with one another). For example, Murray and Bohata played key roles in establishing the university's first dedicated Climate Action Research Institute and Climate Action Research Network (for the Faculties of Science and Engineering and Humanities and Social Science, respectively). Thomas led a Climate Comic project to explore and facilitate intergenerational learning about the CEC (Thomas et al., 2023), and Pigott ran a successful Fossil Free Career campaign with students to persuade the university's career service to cut its ties with fossil fuel employers (Pigott, 2024). It is important to note that these activities were not direct “outputs” of the Climate Lab; the Labs were not intended as spaces to workshop ideas or create action plans. However, they did create the necessary psychological support and community for us, as participants, to feel emboldened to act on our convictions.

The “ripple effects” of Climate Lab resonate with a comment from the influential academic and activist, Charlie Gardner,^9^ that the primary concern of climate-concerned academics should not be to “get people to care about climate change, because they probably already do. Rather, the task is to help them realise their agency, empower them to take action, and facilitate that.” Climate Lab empowered us to first realise *ourselves* as agents of change, making the task of empowering others to do the same feel much more achievable.

**Figure 5:**
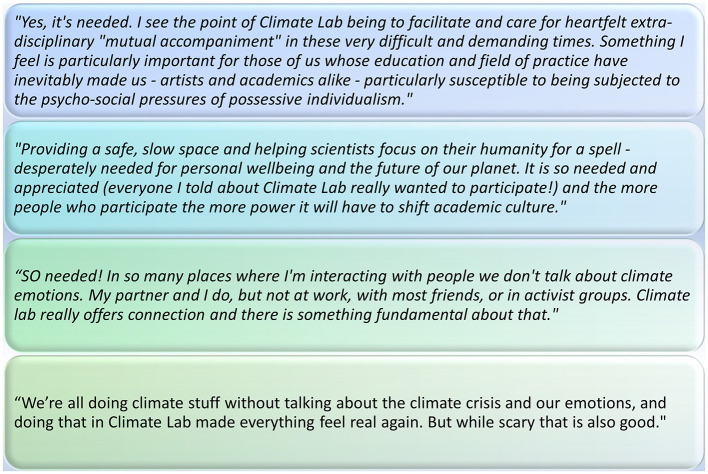
Climate Lab participants, in response to the feedback question(s) “What is the point of Climate Lab? Is it needed? If so, why should we keep offering it?”

Noting these ripple effects (and knowing that many more may have been set in motion by other Climate Lab participants^10^) is important in the context of valid concerns about whether supposedly climate-oriented activities actually contribute to the urgent (and many would argue at this point, primary) task of dismantling fossil capitalism (e.g. Malm, 2018; Bluwstein, 2021), or whether they distract from it. It would be easy to level such claims at Climate Lab, as a space that resists an academic impulse to want to “do” and “solve” (e.g. Stengers, 2018), which takes up time with seemingly frivolous creative activities that may seem self-indulgent in the extent that it delves into the emotions and vulnerabilities of otherwise privileged academics in relative positions of power. As one Climate Lab participant put it, with a nod to the seeming futility of sitting around in a room, talking, “I feel I should be lying down in the road.” However, to write emotional methodologies off because of a lack of immediate or obvious “impact” on fossil fuel industries would be a mistake, and one that is rooted in the particularly linear, positivist, and productivist mindsets that are responsible for a “maladaptive cognitive-practice gap” (Thierry et al., 2023, p. 1) in HE and which are intimately linked to the CEC. As already mentioned, emotional methodologies can help build the deep determination, networks and community cohesion needed to persist in climate actions and can spark inspiration for more visible actions. As participants attest, these spaces and processes are needed (Figure 5). But more than this, engaging in EMs is part of a prefigurative politics (Monticelli, 2022) for post-fossil capitalism worlds, within HE and beyond, in which different kinds of knowledge and embodiment practices (that resist capital-colonialist logics) are prioritised in order to usher in more compassionate, caring, and care-full worlds. As one participant reflected, “working with the artists gave me permission to be playful, to think about and care about my body and emotions, rather than sidelining these. It reminded me of the importance of making contact with some of the ‘softer' or more spiritual aspects of life, even in the face of crises which seem to scream for ‘hard' action.” Prefigurative practices are important not only because they are part of the imaginative work of dismantling fossil capitalism and its logics (imagining that there is, after all, an alternative), but also because they will be part of creating the fairer, more inclusive and more ecologically-sensitive institutions and societies that we desperately need when it is gone.

### 4.4 Emotion, decolonisation, and gender

Transformations towards more sustainable and just futures require a radical dismantling and reconfiguration of long-run sociocultural and political-economic norms currently reproducing the very problems driving climate change, including colonialism, extractivism, neo-liberal capitalism, and an ideology of individualism (Stoddard et al., 2021; Machado de Oliveira, 2021). Western universities are systematically founded on a colonial legacy of knowledge production methods and face an on-going intellectual battle to accept this and to transform their theories, methods, and practices (Ferreira da Silva, 2007; Shilliam, 2014; Todd, 2016). Calls for alternative knowledge creation and meaningful decolonising practices include experiments in trust, communication, deep listening, praxis and reflexivity (Bhambra et al., 2020; Radcliffe, 2017; Smith, 2021; Machado de Oliveira, 2021), which are all facets of emotional methodologies.

One example of the effects of on-going colonial structures during Climate Lab was the use of the English language. We, as organisers, did not even question this choice for the in-person Labs, as English is the dominant working language on campus (although Welsh is also used, and some Welsh was incorporated into the workshops). The use of English also felt unavoidable for the online Global Climate Labs, partly due to the constraints of the language of the organisers and facilitators (predominantly English), but mainly because English remains the international language most likely to enable participants with various first languages to communicate with one another. However, feedback from colleagues when we were promoting Climate Lab indicated that language was a very real barrier to many scientists, especially those who either don't speak English or wouldn't feel confident enough to actively participate and discuss emotions in a room (virtual or otherwise) in which English is the main communication language. To make things worse, in many regions, English is negatively perceived as an imperialist language. These difficulties around language are connected to the ways in which expressions of climate emotions risk perpetuating white, colonial fragility, guilt and inertia (Kanngieser, 2016). As Ray (2021) warns, “Intense emotions mobilise people, but not always for the good of all life on this planet.”

Despite these challenges and potential pitfalls, we argue that utilising emotional methodologies in universities that are structurally and systematically colonial is still a worthwhile endeavour if (a) these methods are facilitated with an awareness of and sensitivity to the colonial nature of universities and to the critical question of *whose* emotions get to count (Ahmed, 2014) and (b) help to build concrete, day-to-day practices and norms that are explicitly decolonial (Kanngieser, 2016)—such as cultures of deep listening, interdisciplinarity, vulnerability, reciprocity, and approaches to knowledge creation that decentre white, masculinised scholarship.

Intersecting with the challenges of coloniality, is the issue of gender. As already noted, the vast majority of Climate Lab participants, as well as its organisers, facilitators, and artists, were female. This initially surprised us (after all, climate science is still dominated by men (Liverman et al., 2022)), but on further consideration is perhaps not surprising at all—and gives cause for concern. Part of the reason that emotional methodologies can bolster decolonial agendas in universities is precisely because they centre qualities such as relationality, care, solidarity, co-operation and attentiveness that do not serve the capitalist, neoliberal values that are increasingly structuring university operations (McGeown and Barry, 2023). Given that women and femme-identifying people are strongly socialised and morally impelled to engage with care-related work (that emotional methodologies might reasonably be classed as) in ways that men are not (see Lynch, 2021, p. 11; also Tronto, 1993 for the nuances around this framing), it was perhaps inevitable that women were more attracted to the premise of Climate Lab, whereas their male counterparts may have seen the invites but prioritised more “valuable” academic activities instead. Equally, the mention of emotions and feelings in the invites may have unintentionally signalled a “female” space and made male-identifying people feel excluded or uncomfortable with participating due to the gender norms that they, too, are constrained by.

The reasons for a lack of male engagement are likely multiple and intertwined, but they are worrisome because it indicates that emotional methodologies, though important for the transformation of HE, risk becoming an additional labour that is predominantly shouldered by women. Women—in HE and elsewhere—are already disproportionately engaged in tending affective relations that require time and proximity (Lynch, 2021) but which are not rewarded within current models of scholarship. What is more, this gender bias is exacerbated by issues of academic rank, race and ethnicity, disability, and employment status (see Owens et al., 2023).

The gender imbalance we observed in Climate Lab is as deeply-rooted in the structures of HE as colonialism is. It may be that a careful rewording of invites to make them less gendered would help, but it is likely that more structural changes in universities that would persuade or enable male-identifying colleagues to take emotional methods more seriously as part of their research and personal development will be necessary. If emotional methodologies are used more widely in HE settings—as we advocate—then it is essential that these problems are addressed so that EMs do not simply further entrench existing gender (and other) inequalities and burden women disproportionately with responding to the CEC—both within HE and more broadly (e.g. United Nations, 2022). With that said, in attempting to address gender inequality it is important not to eschew (in a “throwing the baby out with the bathwater” kind of way) the opportunities and possibilities afforded by feminine knowledge practices (e.g. relationality and emotional reflexivity) and feminist critiques of the status quo (Jaggar, 2014).

## 5 Concluding thoughts

The climate and ecological crises are accelerating, and the need for significant societal change and new ways of acting are critical. This is just as true for the HE sector—which carries a large responsibility to respond to the CEC—as anywhere else. HE is under pressure to act in new ways (e.g. Bhambra et al., 2020; Green, 2020; Facer, 2021; Gardner et al., 2021; Capstick et al., 2022; McGeown and Barry, 2023), but the simplicity of the phrase “act in new ways” belies the deep, often challenging, personal (but socially-determined) changes that support genuinely different ways of working. Learning to act in new ways is unlikely to happen through bolt-on programmes or new toolkits; rather, genuine transformation is a praxis – iterative, difficult, and ongoing. Our central point in this article has been that in order for universities to become agents of change in society through initiating and sustaining “outward” actions (for example, outreach, activism, research initiatives, changing the curriculum, and green infrastructure), there is a need for them to overcome institutionally organised climate silence which is rooted in a denial of climate emotions. Such denial is exemplified by many of the comments by Climate Lab participants in this article, and by the widespread failure of universities to rise to the challenges of the CEC so far.

Our experience in organising and participating in Climate Lab indicates that creating spaces for staff to take a “deep dive” into climate emotions can offer them relief from the cognitive dissonance of suppressing emotions and thus open up new possibilities for, and a determination to sustain, collaborative action with colleagues. Climate Lab also teaches us that artistic and creative methods are invaluable for curating and facilitating such spaces; not as public relations for “Science,” but because they present ways of doing and being that make possible different kinds of knowing and acting. Creative methods help to create the kind of “emotional habitus” needed for staff to feel safe enough and supported when expressing distressing emotions within university environments, helping lead participants away from relying solely on traditional models of climate communication (persuasion, education, and deliberation) and towards models of communication founded on imagination, compassion and respect (Moser, 2015). It follows that once staff feel comfortable with such methods, then they will be better equipped to share these with their students (Owens et al., 2023). In addition to the mental health and climate empowerment benefits of increasing emotional reflexivity amongst staff and students, bringing emotional methodologies “into the fold” as a valid form of knowledge production is also fundamental to the work of decolonisation and gender equality, which are both intrinsically connected to the CEC (e.g. Plumwood, 2002; Smith, 2021; Sultana, 2022).

We know that simply conveying more information, more facts, and more dire warnings about the CEC is not an effective pathway to action, and that engaging people's emotions and imagination is vital for communicating crises and triggering a sense of agency and responsibility (e.g. Guenther, 2020). However, within HE we have been reluctant to heed this advice. It is still—especially within the sciences—relatively “taboo” to acknowledge emotions in and about our research, stemming from deep-seated social norms of Western-Enlightenment science (Brysse et al., 2013). If the climate and ecological crises require those of us working in HE to “dismantle taken-for granted ideas and inherited practices, and to experiment with what a new higher education might be” (Facer, 2021, p. 10) then Climate Lab indicates that engaging with, and making space for, emotions is an essential part of this endeavour.

## Data Availability

The raw data supporting the conclusions of this article will be made available by the authors, without undue reservation.

## Footnotes

^1^https://www.isrf.org/events/conference/ Last accessed 14 June 2024.

^2^One example is the Daring Classrooms initiative (see https://brenebrown.com/hubs/daring-classrooms-hub/ Last accessed 14 June 2024).

^3^See https://fernsmith.uk/ (last accessed 20 June 2024).

^4^See https://www.instagram.com/maregap/ (last accessed 20 June 2024).

^5^See https://climatelab.swansea.ac.uk/climate-lab-swansea/ Last accessed 14 June 2024.

^6^See https://climatelab.swansea.ac.uk/resources/ (Last accessed 14.06.2024).

^7^Available at https://emergenceuk.blogspot.com/2022/04/taking-climate-experts-out-of-thebox_19.html Last accessed 18 March 2024.

^8^It is important to note that, beyond a Padlet set up for participants to share their projects and resources, the Climate Lab did not aim to forge an enduring, active community of connected researchers. Nonetheless, a *sense of community* was created simply by breaking the silence.

^9^In a tweet https://twitter.com/CharlieJGardner/status/1593197570712825857.

^10^Tracking longitudinal “impact” has not been a primary or funded component of Climate Lab, although it would be an interesting next step.
